# Is moral elevation an approach-oriented emotion?

**DOI:** 10.1080/17439760.2016.1163410

**Published:** 2016-05-17

**Authors:** Julie Van de Vyver, Dominic Abrams

**Affiliations:** ^a^ Centre for the Study of Group Processes, School of Psychology, University of Kent, Canterbury, UK

**Keywords:** Affect, altruism, moral beauty, motivation, moral elevation, approach

## Abstract

Two studies were designed to test whether moral elevation should be conceptualized as an approach-oriented emotion. The studies examined the relationship between moral elevation and the behavioral activation and inhibition systems. Study 1 (*N* = 80) showed that individual differences in moral elevation were associated with individual differences in behavioral activation but not inhibition. Study 2 (*N* = 78) showed that an elevation-inducing video promoted equally high levels of approach orientation as an anger-inducing video and significantly higher levels of approach orientation than a control video. Furthermore, the elevation-inducing stimulus (vs. the control condition) significantly promoted prosocial motivation and this effect was sequentially mediated by feelings of moral elevation followed by an approach-oriented state. Overall the results show unambiguous support for the proposal that moral elevation is an approach-oriented emotion. Applied and theoretical implications are discussed.

Researchers have suggested that two systems inform and guide much of our behavior. The behavioral activation system (BAS; Gray, 1982) manages appetitive and approach motivations and behaviors. The behavioral inhibition system (BIS; Gray, 1982) manages aversive, avoidant, and withdrawal-oriented motivations and behaviors. Researchers have typically associated the BAS with positive affect and the BIS with negative affect (Gray, 1982, 1994). Indeed, Carver and White (1994) found that individual differences in BAS were positively associated with individual differences in positive affect, while individual differences in BIS were positively associated with individual differences in negative affect.

More recent evidence shows that negative emotions can also be associated with the BAS. For example, ample research has now shown that anger is high in approach orientation (Carver & Harmon-Jones, 2009; Harmon-Jones, 2007). Therefore, although general negative and positive affect may have quite specific and consistent relationships with the motivational system, individual emotional states (e.g. anger, inspiration, disgust) may have different and more unique relationships with behavioral activation and inhibition systems.

The current research tests whether moral elevation, a relatively under-researched emotion to date, is associated with the BIS or the BAS. More specifically, we test whether elevation should be conceptualized as a high-approach emotion. Researchers have recently begun to explore the nature of moral elevation, in particular focusing on its behavioral outcomes. Moral elevation is a positive emotion experienced when witnessing a virtuous act, one that improves the welfare of others (Algoe & Haidt, 2009). Moral elevation consists of a feeling of warmth and expansion that is accompanied by admiration and affection for the person(s) who performed the exemplary behavior. The action tendency of moral elevation is to emulate the moral exemplar, and to become a better person oneself (Haidt, 2003).

Empirical studies have consistently shown that moral elevation promotes prosocial behaviors (e.g. Schnall, Roper, & Fessler, 2010; Van de Vyver & Abrams, 2015). Prosocial behaviors are those that benefit others (Penner, Dovidio, Piliavin, & Schroeder, 2005). For example, moral elevation promotes charitable donations, volunteering, helping the experimenter, and organizational citizenship (Cox, 2010; Schnall et al., 2010; Van de Vyver & Abrams, 2015; Vianello, Galliani, & Haidt, 2010). Based on this evidence that moral elevation is a positive emotion which produces prosocial behavioral responses, we propose that moral elevation should be moderately or strongly associated with the BAS, and therefore motivate an approach orientation.

A contrasting hypothesis seems plausible on the basis of the one study that has, somewhat indirectly, explored the behavioral activation and inhibition systems of moral elevation. Based on this study the authors suggested that elevation is ‘a calmer emotion which seems to increase openness and warmth towards others; it may not lead to immediate altruistic action when such action is difficult’ (Algoe & Haidt, 2009, p. 30). Moreover, Algoe and Haidt (2009) noted that elevation is associated with a release in oxytocin, which has sedative and stress-reducing effects (Silvers & Haidt, 2008). Algoe and Haidt (2009) coded participants’ open-ended and self-reported motivations and actions following feelings of elevation, admiration, gratitude, or joy. One of the codes was ‘energization,’ which was described as: ‘when people indicated that they wanted to do things like jump up and down, or shout with excitement’ (p. 111). Analyses revealed that admiration and joy involved energization but elevation did not. These findings seem to indicate that elevation may not be strongly associated with the BAS, and may therefore not be considered as an emotion that is high in approach.

## The current research

Algoe and Haidt’s (2009) study used coding of participants open-ended motivational statements as ‘energization’ to conceptualize elevation as an emotion that is low in approach orientation. However, this code seems unlikely to cover all aspects of the BAS. Given this limitation and the contrasting hypotheses based on inferences from prior research, it seems that further research is necessary to more concretely test the relationship between elevation and the behavioral activation and inhibition systems. Study 1 examines the relationship between individual differences in moral elevation and individual differences in the BIS and the BAS, using well-established measures of these constructs. In order to effectively differentiate these relationships, Study 1 also measures general positive and negative affect, as well as individual differences in anger and shame, the former consistently conceptualized as an approach-oriented emotion and the latter as an avoidance-oriented emotion. Given the well-established relationships between general negative and positive affect and the BIS and the BAS (Carver & White, 1994), their effects will be accounted for in regression analyses. Comparing effects of elevation to those of shame (an avoidance-oriented emotion) and anger (an approach-oriented emotion) will help inform the relationships between elevation and the BIS and the BAS. For example, if effects of elevation mirror those of shame, then elevation may be an avoidance-oriented emotion. In contrast, if effects of elevation mirror those of anger, then elevation may be an approach-oriented emotion. If elevation relates to the BAS, but more weakly than anger does, then it may not be a strong approach-oriented emotion. If elevation relates to the BAS more strongly than anger does, then it may be a particularly strong approach-oriented emotion.

Study 2 directly manipulates moral emotions in order to test whether an elevation-inducing stimulus (as compared to an anger-inducing and a control stimulus) increases approach or avoidance orientations, as well as prosocial motivation more specifically.

## Study 1

### Method

#### Participants

Eighty American participants (64.2% female) with a mean age of 37.79 (*SD* = 14.26) completed an online questionnaire (via the Qualtrics software platform). Participants were sampled from Amazon’s Mechanical Turk; a site for web-based data collection that functions through a participant compensation system.

#### Procedure

Participants were given access to a secure web link which sent them to an online questionnaire. Informed consent and demographic information were obtained. Participants then responded to the emotion and motivational orientation measures. Participants received (online) written debrief and compensation upon completion. Participants completed dispositional measures of elevation, anger, shame, approach and avoidance, and measures of current affect.

### Dispositional measures

#### Moral elevation

Moral elevation was measured using a six-item scale taken from Diessner, Solom, Frost, Parsons, and Davidson (2008) (e.g. ‘When perceiving an act of moral beauty I feel changes in my body, such as a lump in my throat, an expansion in my chest, faster heart beat, or other bodily responses’). Participants responded from 1 (*strongly disagree*) to 5 (*strongly agree*). Cronbach’s alpha was .90.

#### Anger

Anger was measured using an 11-item scale taken from Vitaglione and Barnett (2003) (e.g. ‘I feel outraged by injustices done to others’). Participants responded from 1 (*strongly disagree*) to 5 (*strongly agree*). Cronbach’s alpha was .91.

#### Shame

Shame was measured using a four-item sub-scale taken from Cohen, Wolf, Panter, and Insko (2011) (e.g. ‘You make a mistake at work and find out a coworker is blamed for the error. Later, your coworker confronts you about your mistake. What is the likelihood that you would feel like a coward?’) Participants responded from 1 (*very unlikely*) to 7 (*very likely*). Cronbach’s alpha was .76.

#### Approach and avoidance

Approach and avoidance orientations were measured using Carver and White’s (1994) BIS/BAS questionnaire. The BIS (avoidance) scale contains seven items (e.g. ‘I worry about making mistakes’). Cronbach’s alpha for the BIS scale was .86. The BAS (approach) scale consists of three subscales: (1) drive (‘I go out of my way to get things I want’), which contains items that pertain ‘to the persistent pursuit of desired goals’; (2) fun seeking (‘I crave excitement and new sensations’), which has items ‘reflecting both a desire for new rewards and a willingness to approach a potentially rewarding event on the spur of the moment’; and (3) reward responsiveness (‘It would excite me to win a contest’), which contains items that ‘focus on positive responses to the occurrence or anticipation of reward’ (p. 322). Cronbach’s alphas for the three subscales were .86, 76, and 76, respectively. Participants responded to all BIS and BAS items from 1 (*very false for me*) to 4 (*very true for me*).

#### General positive and negative affect

General positive and negative affect were measured using the 20-item positive and negative affect schedule (PANAS, Watson, Clark, & Tellegen, 1988). Participants were asked to rate the extent to which they felt 20 different states (e.g. interested, alert, jittery). Participants responded from 1 (*very slightly or not at all*) to 5 (*extremely*). Cronbach’s alphas were .93 and .95 for positive and negative affect, respectively.

### Results

#### Descriptives

Intercorrelations among variables, as well as their means and standard deviations, are presented in Table 1.

**Table 1.** Study 1: means, standard deviations, confidence intervals, and correlations among key variables.

Variable	Mean (SD)	95% CI	2	3	4	5	6	7
1. Elevation	3.85 (.82)	[3.66, 4.03]	.62^***^	.32^**^	.08	.19	.43^***^	.22
2. Anger	3.94 (.64)	[3.79, 4.08]		.39^***^	−.09	.19	.37^**^	.29^**^
3. Shame	5.87 (1.13)	[5.62, 6.12]			−.24^*^	−.14	.07	.45^***^
4. Approach-drive	2.55 (.70)	[2.40, 2.71]				.57^***^	.54^***^	−.19
5. Approach-fun	2.62 (.65)	[2.47, 2.76]					.48^***^	−.05
6. Approach-reward	3.26 (.49)	[3.15, 3.37]						.13
7. Avoidance	2.98 (.68)	[2.83, 3.13]						

Notes: *N* = 80. CI = confidence interval.

^*^ *p* < .05

^**^ *p* < .01

^***^ *p* < .001.

Correlational analyses showed that moral elevation correlated positively and significantly with BAS-reward (*r* = .43, *p* < .001). Quite similarly, anger correlated positively and significantly with BAS-reward (*r* = .37, *p* = .001) and with BIS (*r* = .29, *p* = .009). Shame correlated negatively with BAS-drive (*r* = −.24, *p* = .030) and positively with BIS (*r* = .45, *p* < .001). There were no other significant relationships between each of the emotions and BIS or BAS. However, when adjusting for Type I error and performing a Bonferonni correction (.05 *α*/6 pairwise comparisons = .008), the relationships between anger and BIS (*r* = .29, *p* = .009) and shame and BAS-drive (*r* = −.24, *p* = .030) disappeared.

#### Regression analyses

Regression analyses were conducted to further examine the relationships between the emotions and the approach and avoidance orientations. Specifically, four sets of regression analyses were conducted to test the predictive effects of each of the emotions on (1) BIS, (2) BAS-drive, (3) BAS-fun, and (4) BAS-reward, while controlling for each other and for general positive and negative affect.

#### BIS

The first regression model was significant, *F* (5, 74) = 13.48, *p* < .001, *R* ^2^ = .48. Shame significantly predicted BIS (β = .33, *p* = .001), while elevation (β = .11, *p* = .332) and anger (β = .18, *p* = .116) did not.

#### BAS-drive

The second regression model was significant, *F* (5, 74) = 3.12, *p* = .013, *R* ^2^ = .17. Shame significantly (and negatively) predicted BAS-drive (β = −.26, *p* = .030), while elevation (β = .18, *p* = .202) and anger (β = −.13, *p* = .365) did not.

#### BAS-fun

The third regression model was not significant, *F* (5, 74) = 1.96, *p* = .095, *R* ^2^ = .12. However, effects mirrored those of BAS-drive, whereby only shame significantly (and negatively) predicted BAS-fun (β = −.27, *p* = .027).

#### BAS-reward

The final regression model was significant, *F* (5, 74) = 5.24, *p* < .001, *R* ^2^ = .26. Elevation significantly predicted BAS-reward (β = .28, *p* = .040), while shame (β = −.11, *p* = .335) and anger (β = .22, *p* = .108) did not.

### Discussion

The findings regarding moral elevation are in line with the hypothesis that it is an approach-oriented emotion. Individual differences in moral elevation were significantly related to individual differences in BAS. Specifically, the higher participants scored on moral elevation, the higher they scored on BAS-reward. Moreover, after accounting for the other emotions as well as general positive and negative affect, moral elevation did not relate to BIS.

Findings for shame are in line with previous research. Individual differences in shame were related to individual differences in BIS (positively) and BAS (negatively). The findings for anger are less clear because the bivariate correlational relationship with BAS-reward became non-significant once other emotions and general positive negative and positive affect were accounted for. These findings are not in line with previous research, which demonstrate that anger is high in approach orientation (Carver & Harmon-Jones, 2009; Harmon-Jones, 2007). The current study did differ from previous research in two ways. First, it measured anger at injustices against others rather than injustices against the self. Second, while previous research has compared anger to positive emotions, this research is the first to compare it to moral elevation. Study 2 will test whether these findings relating to anger are replicated when using an experimental rather than correlational design.

The current findings are consistent with the hypothesis that elevation is an approach-oriented emotion. Indeed, it was the only emotion that positively related to BAS in the regression analyses. However, given the correlational design of the study, it is not possible to establish causal relationships. Therefore, Study 2 employs an experimental design to test the effect of an elevation-inducing stimulus on approach and avoidance orientation, as well as on prosocial motivation more specifically.

## Study 2

Study 2 examines the effects of an elevation-inducing stimulus, as compared to a control stimulus, on approach and avoidance orientations, and on prosocial motivation. As the effects for anger in Study 1 were inconsistent with the existing literature (Carver & Harmon-Jones, 2009; Harmon-Jones, 2007), an anger-inducing condition is included in Study 2 in order to clarify the inconsistencies of Study 1. Furthermore, as Study 1 established that elevation is not an avoidance-oriented emotion, Study 2 does not include shame as a comparison emotion.

Given the results of Study 1, it is hypothesized that the elevation-inducing video will lead to higher levels of approach orientation than the control video. In contrast, levels of avoidance should be low across conditions. Furthermore, it is hypothesized that the elevation-inducing video will lead to higher levels of prosocial motivation than the control video. Finally, the effect of the elevation-inducing video on prosocial motivation should be sequentially mediated by feelings of elevation (rather than anger) and then by the approach-oriented state.

Despite the uncertain conclusions regarding dispositional anger in Study 1, the literature clearly suggests that induced anger should lead to higher levels of approach orientation (Carver & Harmon-Jones, 2009; Harmon-Jones, 2007) and prosocial motivation (Thomas, McGarty, & Mavor, 2009) than the control condition. Furthermore, the effect of the anger-inducing video on prosocial motivation should be sequentially mediated by feelings of anger (rather than elevation) and then by the approach-oriented state.

### Method

#### Participants and design

Seventy-eight American participants (52.60% female) with a mean age of 34.83 (*SD* = 11.02) completed an online questionnaire (via Qualtrics). Participants were sampled from Amazon’s Mechanical Turk; a site for web-based data collection that functions through a participant compensation system. Participants were randomly assigned to condition in a one-factor between-participants design with three levels (condition: elevation, anger, control). Data were collected using an online questionnaire.

#### Procedure

Participants were given access to a secure web link which sent them to an online questionnaire. Informed consent and demographic information were obtained, after which participants were randomly assigned to watch one of three videos (elevation, anger, and control). Participants then responded to the emotion and motivational orientation measures. Participants received (online) written debrief and compensation upon completion.

### Experimental manipulations

#### Elevation

The elevation-inducing video (2.14-min) was taken from Van de Vyver and Abrams (2015) and describes the story of a man (Richard Moore) who had been shot between the eyes with a rubber bullet, which permanently blinded him at the age of 10. It shows how he was able to forgive the perpetrator, take a positive attitude to life, and spend his life helping others.

#### Anger

The anger-inducing video was a 2.58-min video taken from Van de Vyver and Abrams (2015) and demonstrates how a British multinational company dodges taxes in Zambia, and how this affects the people in Zambia (a small-business owner in Zambia pays more a year in tax than the multinational company).

#### Control

As in Van de Vyver and Abrams (2015) a nature video served as the control stimulus (2.42-min) – a National Geographic Channel extract on *Wildebeest Migration*.

#### Measures

All items were measured on a seven-point scale (1 = didn’t feel it at all, 7 = felt it very strongly).

#### Elevation

Participants were asked to rate the extent to which they felt: inspired, awe, uplifted, and admiration (Freeman, Aquino, & McFerran, 2009; Van de Vyver & Abrams, 2015). Cronbach’s alpha was .83.

#### Anger

Participants were asked to rate the extent to which they felt: angry, infuriated, outraged, and contempt (Russell & Giner-Sorolla, 2011). Cronbach’s alpha was .95.

#### Action readiness

Items were drawn from Frijda, Kuipers, and Ter Schure’s (1989) list of action readiness items. We used the items that directly measured approach vs. avoidance orientation. Approach orientation was measured using the following two items: ‘I want to approach, to make contact’ and ‘I wanted to be or stay close, to be receptive to someone’ (*r* = .79, *p* < .001). Avoidance orientation was measured using the following two items: ‘I wanted to have nothing to do with something or someone, to be bothered by it as little as possible, to stay away’ and ‘I wanted to sink into the ground, to disappear from the Earth, not to be noticed by anyone’ (*r* = .61, *p* < .001).

#### Prosocial motivation

Participants were asked to rate the extent to which they felt each of the following things while watching the video: ‘want to help others,’ ‘feel like doing good for others,’ ‘do not want to help others,’ and ‘do not care about assisting others.’ Cronbach’s alpha was .77.

### Results

#### Descriptives

Intercorrelations among variables, as well as their means and standard deviations, are presented in Table 2.

**Table 2.** Study 2: means, standard deviations, confidence intervals, and correlations among key variables.

Variable	Mean (SD)	95% CI	2	3	4	5
1. Elevation	4.13 (1.55)	[3.78, 4.48]	−.18	.41^***^	.01	.36^**^
2. Anger	2.97 (1.98)	[2.53, 3.42]		.42^***^	.36^**^	.24^*^
3. Approach	3.83 (1.78)	[3.43, 4.24]			.21	.62^***^
4. Avoidance	1.93 (1.20)	[1.66, 2.20]				−.15
5. Prosocial motivation	5.70 (1.22)	[5.43, 5.98]				

Notes: *N* = 78. CI = confidence interval.

^*^ *p* < .05

^**^ *p* < .01

^***^ *p* < .001.

#### Emotions

A mixed-model analysis of variance testing the interaction effect of condition (elevation vs. anger vs. control) × emotion self-reports (elevation vs. anger) revealed a significant interaction, *F* (2, 75) = 61.48, *p* < .001, η^2^ = .62. Pairwise comparisons showed that feelings of elevation were higher following the elevation-inducing (*M* = 5.45, *SE* = .24) than following the anger-inducing (*M* = 3.38, *SE* = .23) and control (*M* = 3.56, *SE* = .25) videos (*ps* < .001, respectively), *F* (2, 75) = 22.35, *p* < .001, η^2^ = 37. Feelings of anger were higher following the anger-inducing (*M* = 4.96, *SE* = .25) than following the elevation-inducing (*M* = 2.02, *SE* = .26) and control (*M* = 1.69, *SE* = .27) videos (*ps* < .001, respectively), *F* (2, 75) = 50.77, *p* < .001, η^2^ = .58.

#### Motivational orientation

A mixed-model analysis of variance with motivational orientation (approach vs. avoidance) as a two-level within-participants variable and condition (elevation vs. anger vs. control) as a three-level between-participants variable revealed a significant main effect of motivational orientation, *F* (1, 75) = 81.29, *p* < .001, η^2^ = .52 and a significant main effect of condition, *F* (2, 75) = 8.34, *p* = .001, η^2^ = .18. The main effects were qualified by a significant interaction effect, *F* (2, 75) = 5.27, *p* = .007, η^2^ = .12.

Pairwise comparisons showed that approach orientation was significantly higher following the elevation-inducing (*M* = 4.35, *SE* = .32) and anger-inducing (*M* = 4.39, *SE* = .30) videos than following the control video (*M* = 2.63, *SE* = .33, *p*’s < .001), *F* (2, 75) = 9.78, *p* < .001, η^2^ = .21. There was no significant difference between the elevation-inducing and anger-inducing videos (*p* = .915). Avoidance orientation did not differ by condition (*M*_elevation_ = 1.77, *SE* = .23; *M*_anger_ = 2.29, *SE* = .22; *M*_control_ = 1.69, *SE *= .24; all *p*’s > .072), *F* (2, 75) = 2.01, *p* = .142, η^2^ = .05.

Moreover, pairwise comparisons showed that approach was significantly higher than avoidance following the elevation-inducing (*F* (1, 75) = 51.45, *p* < .001, η^2^ = .41), anger-inducing (*F* (1, 75) = 37.05, *p* < .001, η^2^ = .33), and control (*F* (1, 75) = 6.29, *p* = .014, η^2^ = .08) videos. Nevertheless, the effect sizes for the differences between approach and avoidance orientations were much larger following the elevation-inducing and anger-inducing videos than following the control video.

#### Prosocial motivation

An analysis of variance was conducted to test the effect of condition (elevation, anger, control) on prosocial motivation. Results revealed a significant effect of condition, *F* (2, 75) = 6.03, *p* = .004, η^2^ = .14. Pairwise comparisons revealed that prosocial motivation was significantly higher following the elevation-inducing (*M* = 6.07, *SE* = .23) and anger-inducing (*M* = 5.94, *SE* = .22) videos than following the control (*M* = 5.03, *SE* = .23, *p*’*s* < .007) video. There was no significant difference in prosocial motivation between the elevation-inducing and anger-inducing videos (*p* = .679).

#### Mediation analyses

Sequential mediation analyses (using Hayes, 2013, process macro-model 6) were conducted to test (1) whether feelings of elevation and then approach orientation mediated the effect of the elevation-inducing video on prosocial motivation and (2) whether feelings of anger and then approach orientation mediated the effect of the anger-inducing video on prosocial motivation. Condition (elevation vs. anger vs. control) was dummy coded to produce an elevation condition variable and an anger condition variable. The control condition acted as the reference category. In order to test the independent mediating roles of the two emotions, feelings of anger were entered as a covariate in the first analysis, and feelings of moral elevation were entered as a covariate in the second analysis.

**Figure 1.**
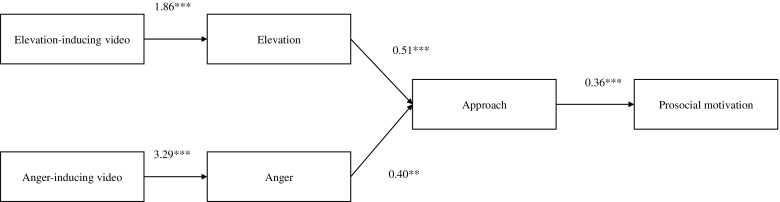
Study 2. Unstandardized *B* coefficients for mediation analyses using Process Macro (Hayes, 2013, model 6).

Results showed that feelings of elevation and then approach orientation significantly and sequentially mediated the effect of the elevation-inducing video on prosocial motivation, *B* = .34, *SE* = .14, 95CI .13/.71 (indirect effect). The significant total effect of the elevation-inducing video on prosocial motivation (*B* = .98, *SE* = .32, *t* = 3.05, *p* = .003) was reduced to non-significance in the direct model (*B* = .23, *SE* = .33, *t* = .69, *p* = .491) (see Figure 1).

**p < .01. ***p < .001.

Similarly, results showed that feelings of anger and then approach orientation significantly and sequentially mediated the effect of the anger-inducing video on prosocial motivation, *B* = .47, *SE* = .24, 95CI .15/1.09 (indirect effect). The significant total effect of the anger-inducing video on prosocial motivation (*B* = .96, *SE* = .30, *t* = 3.16, *p* = .002) was reduced to non-significance in the direct model (*B* = .35, *SE* = .40, *t* = .89, *p* = .378) (see Figure 1).

### Discussion

Results were in line with hypotheses. Specifically, the elevation-inducing and anger-inducing videos led to higher levels of approach orientation than the control video. Moreover, the elevation-inducing and anger-inducing videos led to higher levels of prosocial motivation than the control video. Effects of the emotion-inducing videos on prosocial motivation were mediated by their respective emotional states and then by the approach-oriented state. Therefore, both elevation and anger induce an approach (rather than avoidance) orientation which in turn leads to a greater willingness to help others.

Thus, in line with Study 1, the results of Study 2 provide further evidence for the hypothesis that elevation is an approach-oriented emotion. Furthermore, the results for anger are in line with existing literature (Carver & Harmon-Jones, 2009; Harmon-Jones, 2007) conceptualizing anger as an emotion high in approach orientation.

## General discussion

The current research is the first to directly test the relationship between moral elevation and the behavioral activation and inhibition systems. More specifically, it aimed to explore whether elevation can be conceptualized as an approach-oriented emotion. Study 1 showed that individual differences in elevation (when accounting for general positive and negative affect, and two comparison emotions) were positively associated with individual differences in the BAS (the BAS-reward sub-scale specifically) but not with individual differences in the BIS. This provided evidence for the notion that dispositional elevation is an approach-oriented, rather than avoidance-oriented, emotion. Study 2 showed that an elevation-inducing video, as compared to a control video, significantly promoted an approach-oriented state as well as prosocial motivation. Moreover, the effect of the elevation-inducing video on prosocial motivation was significantly and sequentially mediated by feelings of moral elevation and then by the approach-oriented state. This study showed that situational increases in moral elevation instigate an approach orientation. Taken together the two studies provide clear evidence for the notion that elevation, whether measured dispositionally or as a situational response, is an approach-oriented emotion.

The current findings contribute to the rapidly expanding moral elevation literature. First, the findings somewhat contradict those of Algoe and Haidt (2009). Indeed, elevation does not appear to be a calmer and sedative emotion and instead strongly promotes a desire to do something, to approach, to act, and more specifically, a desire to actively help others. It seems likely that the spontaneously self-described responses coded by Algoe and Haidt may not have captured the full scope of the approach responses that follow from moral elevation. Second, the current findings complement the existing research that has shown the potential of moral elevation for promoting a range of prosocial behaviors (Cox, 2010; Schnall et al., 2010; Van de Vyver & Abrams, 2015), both in the lab and in the field. Specifically, given the approach-oriented nature of elevation, it should effectively promote prosocial behavior both when the behavior is easy as well as when the behavior is difficult. In sum, the findings of the current research provide yet further evidence for the positive and prosocial potential of moral elevation.

### Limitations and caveats

The current research employed anger (Studies 1 and 2) and shame (Study 1) as comparison emotions. These two emotions were employed given their well-established link to approach (Carver & Harmon-Jones, 2009; Harmon-Jones, 2007) and avoidant (Cohen et al., 2011) states, respectively. Future research is now needed to compare the behavioral activation and inhibition systems of elevation to those of other positive emotions that have less obvious action implications (e.g. compassion).

The findings of the current research suggest that elevation is a strongly approach-oriented emotion, and therefore should motivate behavioral responses both when they are easy and when they are difficult (see Algoe & Haidt, 2009). However, the current research did not measure easy vs. difficult prosocial responses and it would be interesting in future research to develop valid and reliable measures of easy vs. difficult prosocial responses to better understand the behavioral limits of different emotions (including elevation).

### Summary

The current research provides clear and direct support for the notion that moral elevation is associated with the behavioral activation rather than inhibition system, and more specifically, that it strongly motivates an approach-oriented rather than avoidance-oriented state. These findings disambiguate contradictions in prior evidence, showing that both dispositional and situational elevation are associated with increased approach. Encouragingly, and in line with past research (Cox, 2010; Schnall et al., 2010; Van de Vyver & Abrams, 2015), these findings suggest that elevation should be a particularly effective emotion for motivating people to proactively help and benefit others.

## Disclosure statement

No potential conflict of interest was reported by the authors.
